# Editorial: Reviews in virus and host

**DOI:** 10.3389/fcimb.2024.1445721

**Published:** 2024-07-08

**Authors:** Gustavo Ramirez-Martínez

**Affiliations:** Laboratorio de Inmunobiología y Genética, Instituto Nacional de Enfermedades Respiratorias, Mexico City, Mexico

**Keywords:** virus, host, evolution, infection, immune response

The intricate interplay between pathogens and humans has traced the evolution of both defense and counteractive mechanisms in both organisms (Kaján et al., 2020), not only drawing our attention to this fascinating race but also giving us insights into the development of public health strategies and policies. The viral life cycle relies heavily on the recognition and interaction of viral proteins with the host cell machinery, mostly with cellular receptors (Grove and Marsh, 2011). Conversely, the host immune system has evolved into a sophisticated set of different types of specialized cells and mechanisms, such as interferon activation, to neutralize viral infection and its clearance (Maarouf et al., 2018). Although both infection and replication mechanisms are similar in the majority of the studied viruses, there is clear evidence of viral heterogeneity in these processes, which is driven by host interactions (Jones et al., 2021). Therefore, further studies about virus and host interactions are necessary to help develop specific antiviral targets and fight emerging viral threats.

In the Research Topic Virus and Host of Frontiers in Cellular and Infection Microbiology, the authors focus the reader´s attention on the relevance of disentangling the mechanisms by which viruses and other microbes counteract host defense mechanisms against infection, along with the evolution of host cellular processes against viral infection.

The rapid adaptive abilities of viruses and other pathogens make their study a difficult task to tackle. With a small genome, viruses have succeeded in leveraging the different complex cellular mechanisms in humans (Durmuş and Ülgen, 2017). Three examples of this are reviewed here. The first involves the impact of several microbes on the copper metabolism of the host. Zhou and Zhang describe the relevance of copper in catalyzing the antimicrobial response by damaging its protein function. The authors review the mechanisms of copper transport, specifically the mechanisms of the copper exporter ATPases ATP7A/B and how some viruses, bacteria, and fungi impact their regulation and localization or use copper pumps to counteract the high copper levels that are detrimental to their survival.

Dong et al. provide another example of how oncogenic viruses, such as the human papillomavirus take advantage of the host cellular mechanisms leading to their genomic remodeling and eventually to cancer development. These oncogenic viruses take advantage of normal cellular processes to modify host gene expression during tumorigenesis and integrate their genetic material into the host DNA (Krump and You, 2018). The authors also review the epigenetic processes induced by these viruses in the host cells and the mechanism of viral protein-host protein interactions, emphasizing how these processes can lead to tumorigenesis.

Autophagy is one of the most described mechanism by which the host immune cells combat viral or microbial infections (Mizushima and Komatsu, 2011). This cellular mechanism is not exempt from viral escape strategies. In their article, Zhai et al. reviewed the complex mechanisms between autophagy and interferon signaling and their relevance in fighting viral infections. The authors emphasize the importance of the PI3K/AKT/mTOR pathway and how autophagy counteracts viral replication through it. They also focus on the NF-κB and eIF2a kinase signaling pathways and how some viruses such as the Influenza A virus counteract by preventing the fusion of autophagosomes or preventing their maturation, thereby promoting the release of viral particles.

Therefore, it is also important to pinpoint the viral countermeasure mechanism in order to increase our knowledge and be ready for potential viral epidemics. This is the case of flaviviral diseases, including dengue virus serotype 1 (DENV1). In their article, Henriques et al., guide us through the asymptomatic outcome of the global spread of DENV1 and how these viruses manage to infect the host in a silent way. Even if there is scarce information about these viruses, the authors draw our attention to the concern of other viral asymptomatic infections, their prevalence, and the urge to study them.

Finally, another successful viral strategy to evade the host immune system involves the use of viral proteins. This is the case of the 2C picornavirus proteins which enhance viral replication. Picornaviruses infect a wide range of organs, such as the nervous system, skin, heart, eyes, and liver, among others. Some of these viruses include enterovirus 71, poliovirus, coxsackievirus, rhinovirus, and hepatitis A virus (Zell et al., 2017). In their review article, Yin et al. highlight the importance of delving into the structure of 2C proteins and their interactions with host proteins. The current knowledge of the biochemical features of the 2C proteins and their structural characteristics has been crucial in understanding the viral infection process and future research will help establish treatment strategies.

Altogether, the Review Articles included here provide a general landscape of some of the host/viral mechanisms used in this endless war. All the authors agree that emphasizing the evolutionary aspects of all these mechanisms will be quite useful in identifying potential threats to human health.

## Author contributions

GR-M: Writing – original draft, Writing – review & editing.
